# Information-Based Principle Induces Small-World Topology and Self-Organized Criticality in a Large Scale Brain Network

**DOI:** 10.3389/fncom.2018.00065

**Published:** 2018-08-07

**Authors:** Kosuke Takagi

**Affiliations:** Independent Researcher, Saitama, Japan

**Keywords:** functional connectome, information processing, mutual information, phase transition, self-organized criticality, small-world network

## Abstract

The information processing in the large scale network of the human brain is related to its cognitive functions. Due to requirements for adaptation to changing environments under biological constraints, these processes in the brain can be hypothesized to be optimized. The principles based on the information optimization are expected to play a central role in affecting the dynamics and topological structure of the brain network. Recent studies on the functional connectivity between brain regions, referred to as the functional connectome, reveal characteristics of their networks, such as self-organized criticality of brain dynamics and small-world topology. However, these important attributes are established separately, and their relations to the principle of the information optimization are unclear. Here, we show that the maximization principle of the mutual information entropy induces the optimal state, at which the small-world network topology and the criticality in the activation dynamics emerge. Our findings, based on the functional connectome analyses, show that according to the increasing mutual information entropy, the coactivation pattern converges to the state of self-organized criticality, and a phase transition of the network topology, which is responsible for the small-world topology, arises simultaneously at the same point. The coincidence of these phase transitions at the same critical point indicates that the criticality of the dynamics and the phase transition of the network topology are essentially rooted in the same phenomenon driven by the mutual information maximization. As a consequence, the two different attributes of the brain, self-organized criticality and small-world topology, can be understood within a unified perspective under the information-based principle. Thus, our study provides an insight into the mechanism underlying the information processing in the brain.

## Introduction

The human brain maintains its performance during perception, cognition, and behavior through information processing in the neuronal networks (Linsker, 1988; Gray et al., 1989; Sporns, 2002; Womelsdorf et al., 2007). Information processing is one of central functions of the brain, which organizes the hierarchical structure of neuronal networks. In particular, integrative processing in the large scale network, which interconnects segregated and functionally specialized regions in the brain (Tononi et al., 1994; Hilgetag and Grant, 2000; Sporns, 2013), is related to cognitive functions such as decision making (Friston, 2010; Clark, 2013; Park and Friston, 2013). In order to achieve efficient performance despite the requirements for rapid and flexible adaptation to changing environments (Bassett et al., 2006; Kitzbichler et al., 2009; Clark, 2013; Park and Friston, 2013; Mnih et al., 2015), information processing in the brain might be optimized (Friston, 2010). Since the brain is spatially limited in its finite volume, it is natural to assume that physical constraints, such as the biological costs, require the brain to optimize its function based on the limited resources (Achard and Bullmore, 2006; Chen et al., 2006; Bassett et al., 2010; Bullmore and Sporns, 2012). Due to this issue, the principles based on the information theoretic quantities, such as free-energy (Friston, 2010) and mutual information (Linsker, 1990), provide formulations, which account for the mechanism underlying the function and structure of the brain. However, understanding the details of the mechanism, and the effect of these principles on structural and functional aspects of the brain networks remains an open issue.

Small-world topology and self-organized criticality are major attributes which facilitate information processing in the brain, yet their relations to the principle of information optimization are still unclear. Recent advances in neuroimaging techniques allow noninvasive observation of anatomical and functional pathways in the brain, leading to elucidation of the network structures and dynamics pattern referred to as the connectome (Sporns et al., 2005; Achard et al., 2006; Bassett and Bullmore, 2006; Shmuel et al., 2006; Hagmann et al., 2008; Greicius et al., 2009; Bullmore and Bassett, 2010; Biswal et al., 2010; Brown et al., 2012). Small-world topology is one of common characteristics of the complex networks that appear in a wide range of phenomena (Watts and Strogatz, 1998; Newmann and Watts, 1999), including the functional connectivity in the brain (Achard et al., 2006; Bassett and Bullmore, 2006; van den Heuvel et al., 2008; van den Heuvel and Sporns, 2011). Due to the abundant existence of hubs and highly connected nodes in the small-world network, it generally achieves robust and efficient information transfer (Albert et al., 2000; Latora and Marchiori, 2001). On the other hand, self-organized criticality provides one attractive hypothesis describing the dynamics state in the brain (Bak et al., 1987; Beggs and Plenz, 2003; Beggs, 2008). Self-organized criticality is described as an emergent property of the system. Specifically, the dynamic systems of interconnected nonlinear elements naturally evolve into a self-organized critical state without any external tuning. Due to successive signal propagation at the large scale observed in the brain, the dynamics of individual units can induce rapid adaptive responses to external stimuli (Kitzbichler et al., 2009; Chialvo, 2010; Tagliazucchi et al., 2012). Based on the fact that small-world topology is an attribute arising in the critical state between random networks and ordered ones, the criticality is considered a major cause of this network attribute. However, the relation between these attributes, which are usually established separately, is not yet clearly understood.

In this study, we show a direct evidence that small-world network topology and self-organized criticality are related by the maximization principle of the mutual information entropy. Targeting the large scale brain network, we investigated the functional connectome constructed from the resting-state functional MRI (fMRI) data, which records activation patterns in brain regions during the resting state, and is expected to describe a common architecture of the human brain (Achard et al., 2006; Bassett and Bullmore, 2006; Fox and Raichle, 2007; Hagmann et al., 2008; van den Heuvel et al., 2008; Greicius et al., 2009; Honey et al., 2009; Biswal et al., 2010; Honey, 2010; van den Heuvel and Hulshoff Pol, 2010; Van Dijk et al., 2010; Hlinka et al., 2011). When conceptualizing the brain as an information processing system, successive patterns of activation and deactivation in different brain regions provide a representation of the processing associated with information transfer. Historically, studies based on measurements of the brain's responses to tasks or stimuli have been successful in mapping specific cognitive functions onto distinct brain regions (e.g., Kanwisher et al., 1997). However, accumulated evidence in recent studies indicates that various cognitive functions arise from the more complex dynamics of interactions between distributed brain regions, rather than from activities localized to specific regions (Ghazanfar and Schroeder, 2006; Bressler and Menon, 2010). Further evidences indicates that these activities are efficiently modulated by brain regions that are negatively correlated to tasks and are active and demonstrate spontaneous neural activity even in the resting state (Fox et al., 2005; Menon and Uddin, 2010). Then optimization of information processing is accomplished by coordinating activation and deactivation in different brain regions. Thus, activation correlations and anti-correlations between regions, which are calculated based on resting-state fMRI observations, provide basic information useful in understanding the above processes (Fox et al., 2005; Fox and Raichle, 2007; Uddin et al., 2009).

In our study, we use the preprocessed functional connectome data consisting of a matrix, each element of which represents the connectivity strength between regions (Biswal et al., 2010; Brown et al., 2012). We analyze these data using topological and statistical methods (Barrat et al., 2004; Achard et al., 2006; Clauset et al., 2009; Takagi, 2010, 2017; Klaus et al., 2011). Based on the information transfer model reflecting the topological and functional aspects, we show that the requirement for the maximization of the mutual information entropy drives the network to the critical state. We then show that the phase transition, with respect to the topological structure, appears according to this maximization. Further, we show that, at this critical point, the distribution of the connectivity strength converges to the model, indicating the self-organized criticality (Takagi, 2010, 2017). These evidences describe their relations explicitly, and indicate that they are essentially rooted in the single phenomenon driven by the maximization of the mutual information entropy. Thus, according to our results, the two different attributes of the brain, self-organized criticality and small-world topology, can be understood within a unified perspective, under the information-based principle.

## Materials and method

### Functional connectome datasets

The functional connectome provides a description of the large scale network structure in the brain with the connectivity matrix, whose (*i, j*) element represents the connection weight *w*_*ij*_. The weight *w*_*ij*_ was evaluated from the fMRI data by the correlation coefficient, where each node, *i* or *j*, corresponds to the single region segmented in the brain (Achard and Bullmore, 2006; Achard et al., 2006). In this study, we used preprocessed datasets of the connectivity matrix, which are directly available at the USC Multimodal Connectivity Database (Brown et al., 2012) from the web page (http://umcd.humanconnectomeproject.org/). These matrix datasets have *N*×*N* elements (*w*_*ij*_), which correspond to the connectivity strengths between *N* = 177 brain regions in this case, which are sufficiently large to cover the entire brain (Brown et al., 2012). The matrix datasets used in this study thus contain 986 matrices constructed from data from different individual subjects. They are constructed from the datasets of the functional connectome of “1,000 connectome project” (Biswal et al., 2010), which collects the data obtained by resting-state fMRI (R-fMRI) of the brain. They reveal that, while individual differences can be observed, the connectome datasets share a common architecture.

Because the correlation coefficient indicates a linear relationship between variables, there would be limitations in applying this quantity to brain activity, which is nonlinear. However, it has been reported that resting-state fMRI data are almost Gaussian. As such, the loss in connectivity information due to the use of linear correlation is relatively small (Hlinka et al., 2011). We thus use this quantity, which approximately represents the brain network.

#### Network description

The connectivity matrix contains the noise and artifacts (Eguiluz et al., 2005; Brown et al., 2012; Takagi, 2017), and subsequent noise reduction procedures are required to depict the network structure accurately. During usual analysis, these noises were removed by applying the threshold value to the matrix (*w*_*ij*_). In this process, connections with small connectivity weights are removed, and the network was constructed by the residual connections. Further, this procedure is relevant to the brain network analysis, because it extracts core structures consisting of strongly connected pathways (Hagmann et al., 2008).

Introducing the threshold *w*_*t*_ for the connection weight *w*_*ij*_, we obtained the network description consisting of the connections corresponding to the |*w*_*ij*_| > *w*_*t*_ elements. Since responses of neuronal activity can be categorized as positive and negative ones (Shmuel et al., 2006), *w*_*ij*_ takes its value in the positive and the negative range accordingly, and then we adapted the threshold to the absolute value |*w*_*ij*_|.

This process simultaneously produces the topological description, which was defined by the adjacency matrix (Eguiluz et al., 2005; Bullmore and Bassett, 2010; Honey, 2010). In this matrix, each element *a*_*ij*_ was assigned the binarized value, 0 or 1, according to the absence or presence of the connection between nodes *i* and *j*. For the introduced threshold, the adjacency matrix takes *a*_*ij*_ = 1 for the (*i, j*) element with |*w*_*ij*_| > *w*_*t*_ and *a*_*ij*_ = 0 otherwise.

We use the largest connected component and the clustering coefficient, which are basic measures of the topological network, to characterize the structure of the topological network. For a given graph description, such as that presented above, which is an undirected topological graph based on the adjacency matrix, connected components are defined by connected subgraphs. In each of these subgraphs, all of the vertices are connected to each other by paths. We measure the size of each connected component using the number of vertices in the subgraph. We then determine the largest connected component. In this paper, we measure this quantity using R-package igraph (Barrat et al., 2004). However, the clustering coefficient *C*, which is also known as transitivity, is used to measure the probability that the adjacent vertices of a vertex are connected (Watts and Strogatz, 1998). This quantity provides an important indicator of the small-world network. Unlike networks such as random or regular networks, small-world topology is defined as a network that can be highly clustered into regular lattices, yet have small characteristic path lengths, as in random graphs (Watts and Strogatz, 1998). In our previous work (Takagi, 2017), simultaneous emergence of a small average minimum path length and a large clustering coefficient were observed in the datasets, which we also use in this study. We thus measure the clustering coefficient *C* in this paper as an indicator of small-world topology using R-package igraph (Barrat et al., 2004).

### Information transfer model

On the brain connectivity map represented by the connectivity matrix (*w*_*ij*_) and the corresponding adjacent matrix (*a*_*ij*_), information processing was represented by the signal transmission. Information transfer in the brain can be described by successive propagation of the signal represented by the activated state of each site (Bak et al., 1987; Beggs and Plenz, 2003; Beggs, 2008). In order to model the information transfer, we defined the stimulus signals *S* = (*s*_1_, …, *s*_*N*_) and the responses *R* = (*r*_1_, …, *r*_*N*_), assigning the three states for each *i*-th node *s*_*i*_, *r*_*j*_ ∈ {1, −1, 0} for the network size *N*. The inactivated regions were assigned the 0 state, while the two states at ±1 for *s*_*i*_, *r*_*j*_ were considered to represent positive and negative activations, respectively, in accordance with the empirical fact that responses of neuronal activity can be categorized as positive and negative (Shmuel et al., 2006).

In our simulation, where we use the same probability for positive and negative activation, we assigned 1 and −1 to each input signal *s*_*i*_ with the probability *p*, respectively. This value was set to 0 otherwise. This parameter indicates the strength of activity, which is related to energy consumption in the brain. Because brain activity fluctuates, the activation density is variously taken.

For a given set of signals *S* = (*s*_1_, …, *s*_*N*_) with randomly assigned values of *s*_*i*_, we estimated the response *r*_*j*_ using the total input signals received by each *j*-th node as

(1) $$\begin{array}{l} r_{s,j}=\sum_{i\in N}a_{ij}w_{ij}s_{i},\;(r_{j}=1(r_{s,j}>w_{t}),r_{j}=-1(r_{s,j}<-w_{t}),\\ \;r_{j}=0(w_{t}\geq |r_{s,j}|)) \end{array}$$

where *w*_*ij*_ is the connectivity between *i* and *j*, given by the connectivity matrix, and *a*_*ij*_ is the adjacency matrix. The state of each responding node *r*_*j*_ was determined according to *r*_*s, j*_ and the threshold *w*_*t*_.

### Mutual information entropy

The mutual information entropy is one indicator which measures the information transfer from imposed stimuli to responses. It is frequently used to evaluate the information transfer in networks, including the neural network models and the real brain regions (Bak et al., 1987; Beggs and Plenz, 2003; Beggs, 2008). Therefore, we used this quantity to assess the efficiency of the information transfer in our model.

For the set of stimulus signals *S* and the corresponding responses *R*, the mutual information was defined as *H*(*R*)−*H*(*R*|*S*), where *H*(*R*) is the information entropy of the response *R* and *H*(*R*|*S*) is the conditional entropy. Especially for the transfer between *i* and *j* nodes, the mutual information entropy was estimated by the following equation

(2) $$\begin{array}{l} m\left(i,j\right)=H\left(s_{i}\right)+H\left(r_{j}\right)-H\left(s_{i},r_{j}\right), \end{array}$$

where the entropy, *H*(*s*_*i*_) and *H*(*r*_*j*_), and joint entropy, *H*(*s*_*i*_, *r*_*j*_), were calculated using the probabilities for each state *s*_*i*_, *r*_*j*_ ∈ {±1, 0}. Specifically the entropy *H*(*s*_*i*_) is defined as $H\left(s_{i}\right)=-\underset{s_{i}=\pm 1,0}{\sum }p\left(s_{i}\right)\ln\;p\left(s_{i}\right)$ for *p*(*s*_*i*_), which indicates the probability of the state ±1, 0 at the *i*-th node. *H*(*r*_*j*_) is thus defined by substituting *r*_*j*_ into *s*_*i*_. In addition, *H*(*s*_*i*_, *r*_*j*_) can be calculated using $H\left(s_{i},r_{j}\right)=-\underset{s_{i}=\pm 1,0,r_{j}=\pm 1,0}{\sum }p\left(s_{i},r_{j}\right)\ln\;p\left(s_{i},r_{j}\right)$ for *p*(*s*_*i*_, *r*_*j*_), which represents the joint probability for the combination of *s*_*i*_ and *r*_*j*_ states.

This definition, Equation (2), provides the mutual information entropy for each node, with averaging, as $<m\left(j\right)>=\left(\underset{i}{\sum }m\left(i,j\right)\right)/\left(N-1\right)$ for all the possible connections. Finally, this quantity for the whole network was estimated as $m=\underset{j}{\sum^{\text{​}}}<m(j))>/N$. In this study, we estimated the mutual information entropy according to this definition of the average. The optimal state with respect to information transfer is thus obtained by maximizing this quantity.

## Result

### Mutual information entropy

We calculated the mutual information entropy according to the model represented by Equation (1) and the definition Equation (2). In order to reduce noise and define the weight matrix (*w*_*ij*_) and adjacent matrix (*a*_*ij*_), we introduced the cut-off threshold *w*_*t*_. Considering the differences between individuals, we defined the threshold value *w*_*t*_ based on the average of the connectivity <  |*w*| > and the standard deviation σ_|*w*|_ for each connectivity matrix. We calculated <  |*w*| > and σ_|*w*|_, and defined the cut-off threshold by

(3) $$\begin{array}{l} w_{t}=<|w|>+n\cdot \sigma_{\left|w\right|} \end{array}$$

with a parameter *n*. Further, as explained in the previous section, in order to control the activation density of the input stimuli *S*, we introduced the activation probability *p*, due to which each node is randomly activated.

**Figure 1:**
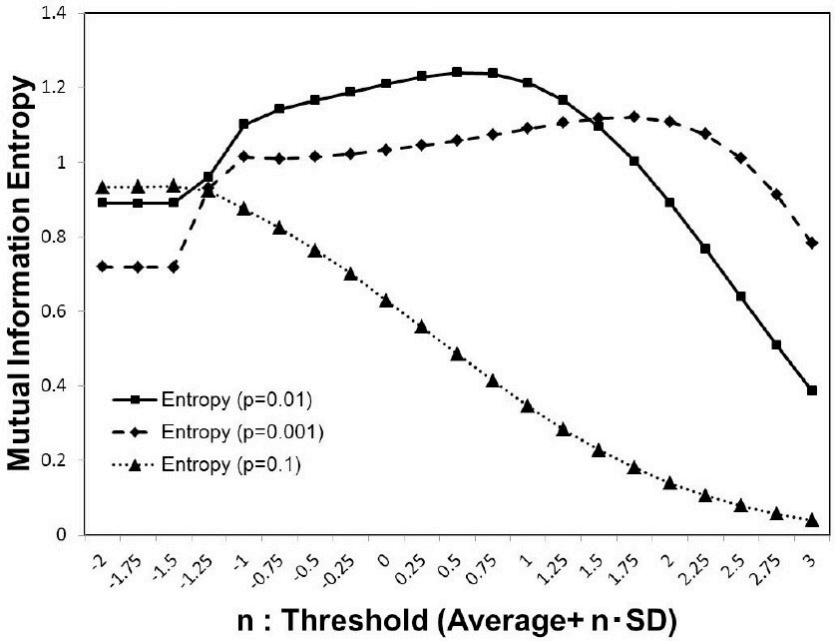
Mutual information. We calculated the average value of the mutual information Equation (2) using whole 986 datasets of the functional connectivity matrices (Biswal et al., 2010; Brown et al., 2012). The threshold value was considered as Equation (3) parameterized by *n* with the standard deviation. We used three different values of the activation probability, *p* = 0.001, 0.01, 0.1, corresponding to the dashed line, solid line, and dotted line on the plot, respectively. Each simulation, was repeated 1, 000 times, with random input signals.

As shown in Figure 1, for each activation probability *p* = 0.1, 0.01, 0.001, we estimated the average of the mutual information entropy for different cut-off threshold values, which were defined as Equation (3). Comparing the peak values for these *p*-values, the maximum value was recorded in the case of *p* = 0.01, with the medium density, while for other cases lower peak maximum values were recorded. The result in Figure 1, showing the three different conditions, indicates that the density of the signal activation is one of the major factors that determine the efficiency of the information processing.

### Largest component size and phase transition

In order to determine other factors which contribute to the increase in the mutual information entropy, we evaluated one of the basic measures of the network, the size of the largest connected component. It is expected that the decomposition of the connected network decreases the mutual information entropy, because the information transfer between separated components is completely prohibited.

**Figure 2:**
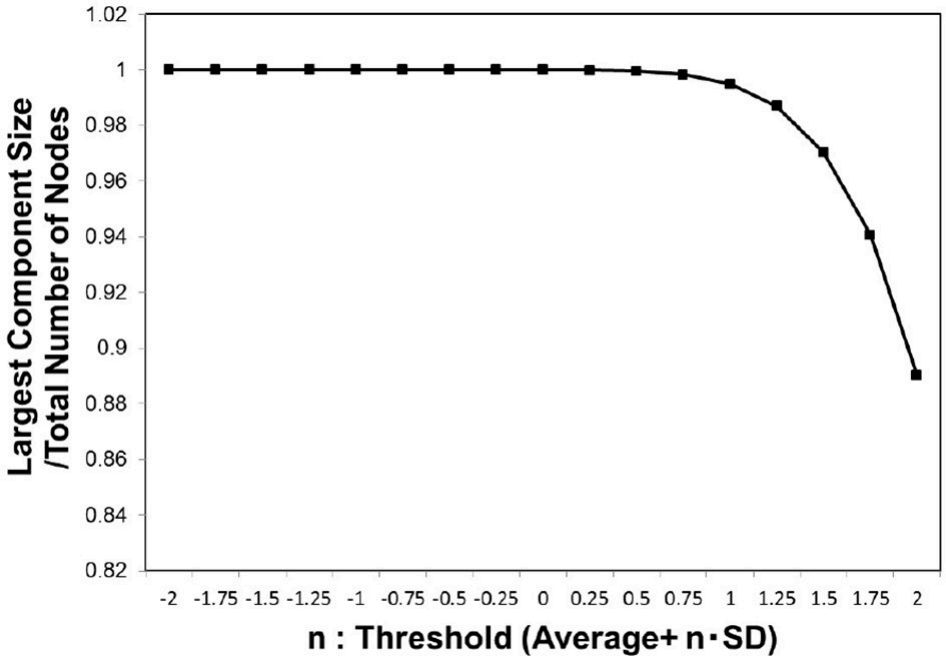
Largest component size. we estimated the largest component size of the topological network representation obtained for the threshold *w*_*t*_ values defined as Equation (3). The vertical axis indicates *n*, which parameterizes the threshold as shown in the definition of Equation (3). The largest component size in the horizontal axis was normalized by the total number of the nodes, and then the value at 1 corresponded to the fully connected network. The average of this value was considered for the whole datasets, 986 datasets of the functional connectome (Biswal et al., 2010; Brown et al., 2012).

We then evaluated the largest component size against the threshold (Figure 2) for whole individual datasets of the functional connectome. In this figure, the size was normalized by the total number of the nodes, and 1 indicates that the network is fully connected. For the adjacent matrix obtained by adapting these threshold values defined as Equation (3), we measured the size of the largest connected component.

**Figure 3:**
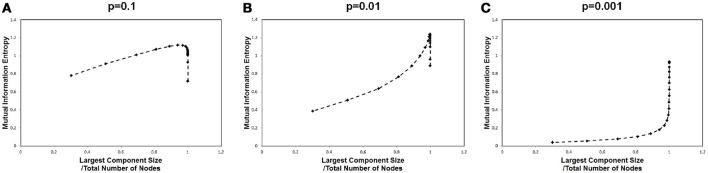
Mutual information and the largest component sizes. We plotted the mutual information value against the corresponding value of the largest component sizes. The mutual information data is the same as that in Figure 1, and the largest component sizes were taken from Figure 2 for each corresponding threshold value, where the largest component size was divided by the total number of nodes. **(A)** The estimated values for a high signal density (*p* = 0.1) are shown. **(B)** The estimated values for a medium signal density (*p* = 0.01) are shown. **(C)** The estimated values for a low signal density (*p* = 0.001) are shown.

In order to identify the relation between the maximization in Figure 1 and the largest component size, we plotted the mutual information values against the corresponding largest component sizes for each cut-off threshold in Figure 3. A sharp peak with a discontinuous curve was observed for the *p* = 0.01 case (Figure 3B), whereas gradual changes appeared in the other cases with *p* = 0.001 and *p* = 0.1 (Figures 3A,C). This behavior in Figure 3B indicates that the largest component size is the other major factor which affects the mutual information entropy. Further, it implies that the maximization of the mutual information is related to the occurrence of the phase transition with respect to the topological structure. The existence of the phase transition observed in Figure 3B might agree with the argument that the brain operates near the critical state (Bak et al., 1987; Beggs and Plenz, 2003; Beggs, 2008; Kitzbichler et al., 2009; Chialvo, 2010; Tagliazucchi et al., 2012).

This maximization might be explained by the criticality hypothesis (Bak et al., 1987; Beggs and Plenz, 2003; Beggs, 2008), which states that the information transfer is maximized in the critical state. This state is in contrast with the sub-critical state with less activation and the super-critical state, in which excess activation is saturated. In sub-critical state, due to poor sensitivity to the stimulus, activations die out, and the signal transfer is terminated quickly. On the other hand, in the super-critical state, the system reaches the runaway excitation due to uncontrolled chain reactions. Therefore, the information transmission is expected to be maximized in the critical state. The result in Figure 3, showing the three different conditions, indicates that the medium density with *p* = 0.01 (Figure 3B) represents the critical state.

### Small-world topology and phase transition

In the above results, we showed that network topology is one of the factors which contribute toward maximization of the mutual information entropy, and this is accompanied by its phase transition. In order to specify the relation between the mutual information maximization and the network topology, we investigated the behavior of the network topology around the critical point in greater detail.

The small-world topology is one of common characteristics of the complex network which arises in the critical state between random networks and ordered ones (Watts and Strogatz, 1998; Newmann and Watts, 1999). Generally, it contributes to the robustness and efficiency in the information transfer in various types of complex networks. It is considered that the small-world architecture is relevant for understanding the function of the brain, and the empirical evidences support this argument (Achard et al., 2006; Bassett and Bullmore, 2006; van den Heuvel et al., 2008; van den Heuvel and Sporns, 2011).

**Figure 4:**
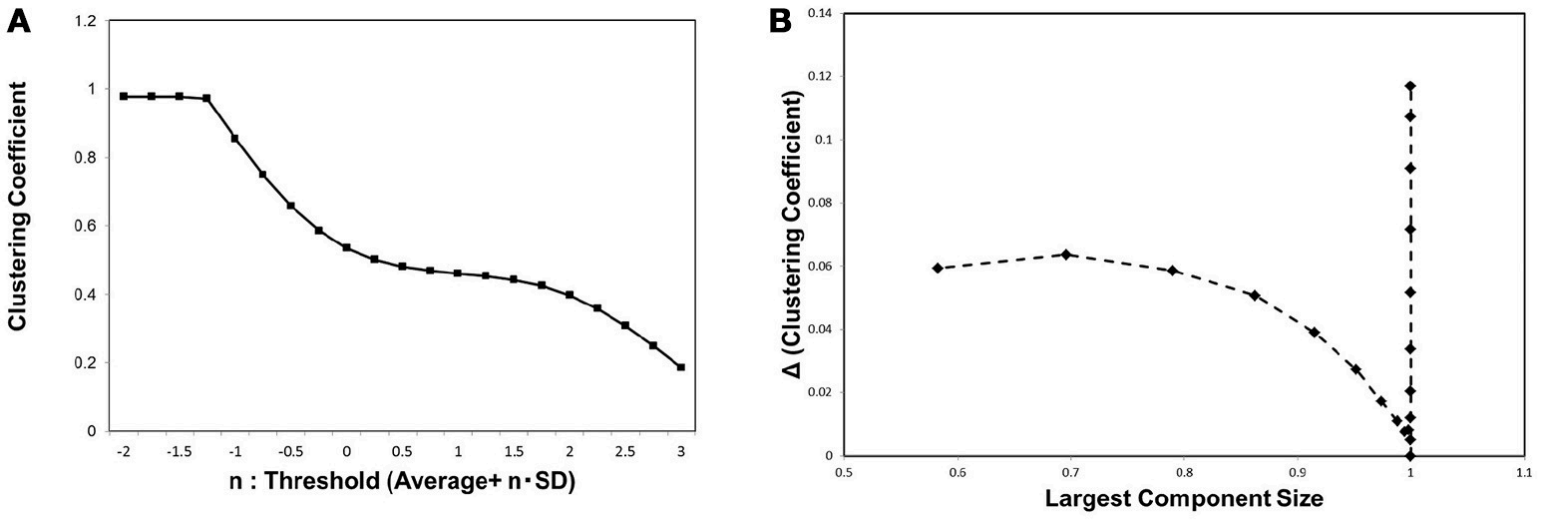
The clustering coefficient and the threshold. **(A)** We measured the clustering coefficient for each threshold value considered as Equation (3). The average was calculated for the whole datasets, 986 datasets of the functional connectome (Biswal et al., 2010; Brown et al., 2012). **(B)** We plotted the changes in the clustering coefficient, Δ*C*, against the corresponding value of the largest component sizes same as shown in Figure 3. The difference in the clustering coefficient Δ*C* was defined as Δ*C* = *C*(*i*)−*C*(*i*+1), where *i* indicates the *i*-th value of the threshold in the panel (A), which is calculated from the minimum value *n* = −2 as (*i* = 0).

In order to characterize the behavior of the network topology around the critical point, we evaluated the clustering coefficient *C*. As explained in the previous section, this basic quantity is frequently used to characterize the small-world network, which exhibits relatively large clustering coefficient values (Watts and Strogatz, 1998). In Figure 4A we show the result of measuring the clustering coefficient. For different threshold values, the clustering coefficient remains almost constant at its value around the critical point specified in Figure 3B. This stability agrees with the observation in the Watts-Strogatz model that the clustering coefficient is stable near the state of the small-world topology (Watts and Strogatz, 1998). The small change in the clustering coefficient around the critical point shows that it has relatively large values during this transition. This explains why the small-world topology appears around this critical point.

In order to provide the further evidence for the relation between small-world topology and phase transition, we measured the changes in this value Δ*C*, and plotted these values against the corresponding largest component size, same as in the case of Figure 3B. Δ*C* was defined by the difference of *C* between the values for neighboring thresholds, Δ*C*(*i*) = *C*(*i*) − *C*(*i* + 1) for the *i*-th threshold value in our calculation. Exhibiting similar behavior to Figure 3B, the plot in Figure 4 specifies the critical point with a sharp peak at the same critical point of the mutual information entropy. Thus, there exists a phase transition regarding the network topology, which is responsible for the small-world feature, and we suggest that this phase transition contributes to the maximization of the mutual information entropy.

### Activation pattern and the self-organized criticality

In our model Equation (1), the other factor, which mainly contributes to the information transfer, is the connectivity strength *w*_*ij*_. The distribution of *w*_*ij*_ is important for controlling the response, especially for hub nodes. On these nodes, the response to signals received from multiple sites is determined according to the combination of *w*_*ij*_, (*w*_*i*_1_*j*_, *w*_*i*_2_*j*_, … ) for *i*_1_, *i*_2_, … . In these responses, highly weighted connections, which organize the core network in the brain, are dominant. The distribution of the connectivity strength is another important factor which determines the efficiency of the information transfer.

In order to describe the contribution of *w*_*ij*_ to the maximization of the mutual information entropy, we identified the statistical characteristics of *w*_*ij*_ around the critical point, and clarified its relation to the criticality observed with the mutual information entropy. For this purpose, we assessed the distribution of *w*_*ij*_, whether it obeys the prediction of the self-organized criticality. In this state, it is predicted that characteristic scales will disappear, and the systems will behave independently of the scale (Bak et al., 1987). The emergence of the power law distribution is considered a typical characteristic observed in this state.

However, when we adapted the power law to the distribution of *w*_*ij*_, the straightforward application was prohibited due to the upper and lower limits of its definition of the correlation coefficient. We then used the distribution model derived from the power law, adapting it to the restricted variable range (Takagi, 2010, 2017). In accordance with the restricted region |*w*| ≤ 1, we applied the power law to the variable $\tilde{w}=\left(1-|w|\right)$, and obtained the expression $p\left(\left|w\right|\right)\propto {\left(\tilde{w}\right)}^{\gamma }={\left(1-|w|\right)}^{\gamma }$, with a constant γ. Normalizing $\int_{0}^{1}drp\left(\left|w\right|\right)=1$ yields the expression of our distribution model,

(4) $$\begin{array}{l} p\left(\left|w\right|\right)=\left(\gamma +1\right){\left(1-|w|\right)}^{\gamma }. \end{array}$$

In order to verify that the distribution follows this model, we assessed the performance of the distribution fitting using the Kolmogorov-Smirnov (KS) distance (Clauset et al., 2009; Klaus et al., 2011). For the cumulative distribution *P*_*e*_(*w*), which is experimentally given, and the model distribution *P*(*w*) fitted to the data, the KS distance *D* is defined as

(5) $$\begin{array}{l} D=\underset{w}{\max}|P_{e}\left(w\right)-P\left(w\right)| \end{array}$$

which measures the maximum distance of the model from the experimental data.

**Figure 5:**
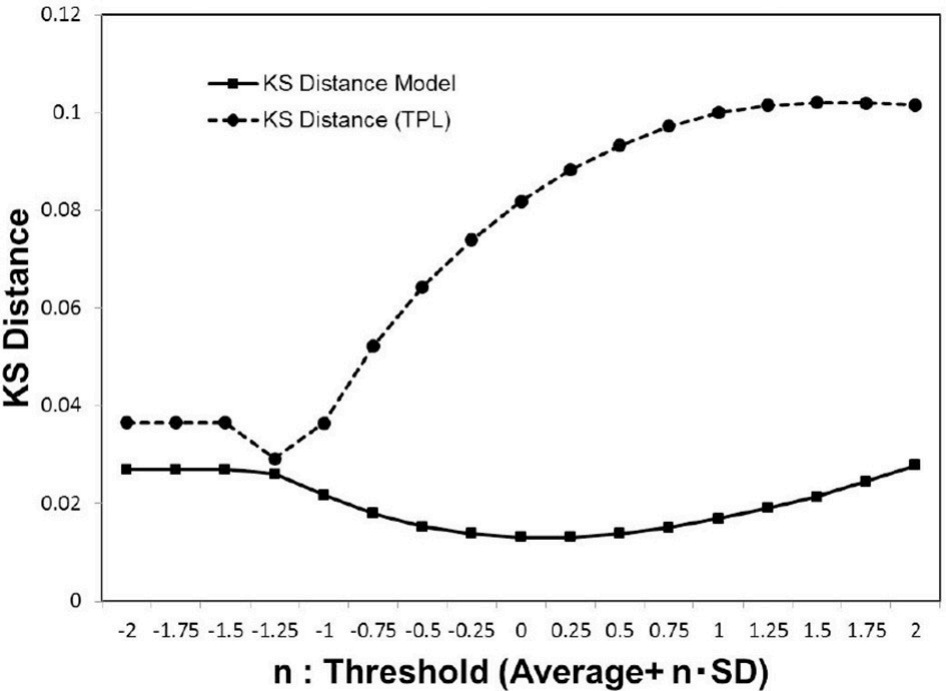
Kolmogorov-Smirnov distance of the distribution models. We estimated the Kolmogorov-Smirnov (KS) distance for each cut-off threshold value. For the cumulative distribution *P*(|*w*|) of the experimental data *w*_*ij*_ which satisfies |*w*_*ij*_|>*w*_*t*_, the parameters of the truncated power law and our models were estimated by the maximum likelihood method for each model. We then estimated the values of the KS distance for the whole datasets (Biswal et al., 2010; Brown et al., 2012), according to the Equation (5), and calculated the averages.

In Figure 5, we show the KS distance values for the noise-reduced weight matrix (*w*_*ij*_), applying the cut-off threshold Equation (3). In the distribution fitting, the parameters of each distribution model were estimated by the maximum likelihood method. This was compared to the truncated power law, which is applied instead of the power law in most cases when the distribution has the upper limit (Achard et al., 2006). The exponentially truncated power law is described as $p\left(x\right)\propto x^{\alpha -1}e^{x/x_{c}}$, where α is a constant exponent, and *x*_*c*_ is the truncation value or the cut-off. For the truncated power law, the maximum likelihood was estimated using R and the R-package brainwaver (http://cran.r-project.org/web/packages/brainwaver) (Achard et al., 2006).

As indicated by the plot of Figure 5, our model yields more stable lower values stably than the truncated power law model. Consequently, our model Equation (4) provides a good fit for the distribution of (*w*_*ij*_). Further, the convergence to this distribution model is indicated by the sufficiently small value of its minimum distance.

**Figure 6:**
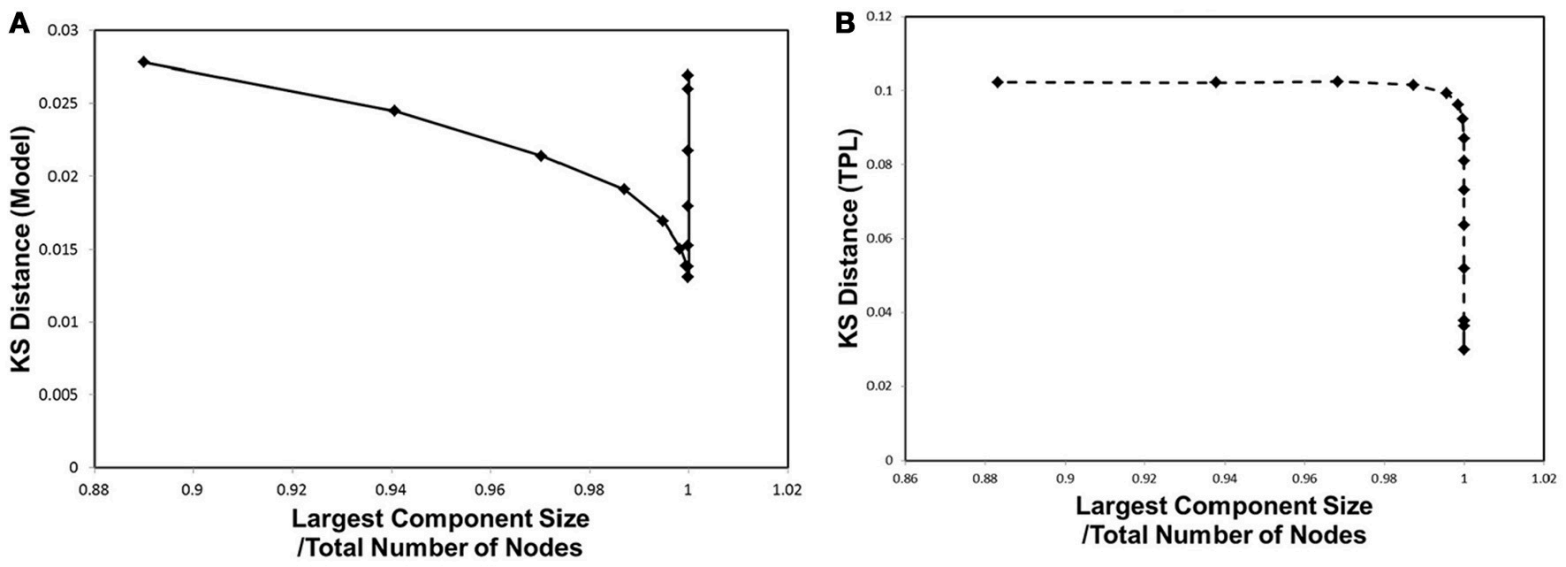
Kolmogorov-Smirnov Distance and phase transition. The Kolmogorov-Smirnov (KS) distance was plotted against the largest component size, same as shown in Figures 3, 4B. **(A)** The KS distance and the largest component size for our distribution model, for *w*_*ij*_, are shown. **(B)** The KS distance and the largest component size for truncated power law distribution are shown.

In order to correlate to the phase transitions shown in Figures 3B, 4B, we combined Figures 2, 5 into Figure 6, plotting the KS distance value against the corresponding largest component size for each cut-off threshold. The resulting distribution model (Figure 6A) exhibits a similar behavior to the cases of Figures 3B, 4B. The plot shows a sharp peak around the critical point, at which point the phase transitions observed with the mutual information entropy and the topology appear.

In comparison with the results of the truncated power law distribution (Figure 6B), the behavior of our model (Figure 6A) clearly exhibits the characteristics of the phase transition with a sharp peak, and depicts its difference with the case of the truncated power law, in which such a peak is absent. In this figure, the distance of the truncated power law increases with that of *w*_*t*_, indicating that the difference from the experimental data becomes significant almost monotonically with decreasing noise. On the other hand, our model shows a decrease toward its minimum peak around the critical point. The presence of the sharp peak is a characteristic behavior observed only in our distribution model.

## Discussion

### Topology and dynamics patterns under the maximization of the mutual information entropy

In this paper, we showed that, due to the maximization of the mutual information entropy in the large scale brain network, small-world network topology and criticality in the activation dynamics are induced. Our simulation results shown in Figure 3B indicate that the requirement for this maximization drives the network state to the critical point specified by the peak of this entropy.

Similar behavior was observed with the clustering coefficient (Figure 4B), indicating that the same mechanism induces the phase transition of the topological structure. This phase transition is responsible for the small-world topology, because this feature emerges during the phase transition between random and ordered networks. Further, the relation to the small-world topology is supported by our result (Figure 4A) showing the small change of the clustering coefficient around this point, indicating that the network has relatively high transitivity at this point.

In addition, this accompanies the emergence of self-organized criticality in the dynamics. This is shown by the convergence of the coactivation pattern distribution to the model, indicating self-organized criticality (Figure 6A). Toward the critical point specified in Figures 3B, 4B, the separation distance between the empirical data and the distribution model measured by the KS distance rapidly decreased. The criticality of this state was confirmed by the fact that this distribution model was directly derived from the power law, one of the characteristic features of self-organized criticality.

These results provide evidence to support that the principle of the mutual information maximization predominantly affects the structural and functional aspects of the brain network. Thus, our results explain the origin of the important attributes of topology and dynamics of the functional connectome.

### Criticality

Our results provide a unified perspective of the topological and functional aspects of the connectome, under the concept of criticality. In Figure 1, we showed three different state, which corresponded to the sub-critical state with low signals (*p* = 0.001), the critical state with the medium signals (*p* = 0.01), and the super-critical state with high signals (*p* = 0.1). The criticality is explicitly shown by the result Figure 3B, in which the phase transition exhibited a sharp maximum peak of the mutual information. At this point, the mutual information entropy was maximized, and subsequently the optimal state, with respect to information transfer, appeared.

This criticality observed with the mutual information entropy explains the origin of the small-world topology and the criticality of the coactivation patterns. As represented in Equation (1), the information transfer depends on the topological structure represented by the adjacent matrix (*a*_*ij*_) and the weight matrix of the connectivity strength (*w*_*ij*_). As indicated by Figure 4B, the critical point of the clustering coefficient, one of the representative topology measures, coincides with that of the information entropy shown in Figure 3B. The maximization of the mutual information entropy induces the phase transition in the network topology. This small-world network contributes to the efficiency of the information transfer, because it contains hubs or highly connected nodes, which have the advantage of shortening the path length between the nodes.

These hubs, which have relatively large number of connections, have an opposite effect of inhibiting efficient communication. The signal transfer model Equation (1) implies that excess signals, which simultaneously reach a single hub node, confuse the transfer and produce noises. It is expected that, for these noises, the connectivity weight extracts the important signals, and then controls the information transfer, while avoiding confusions. The similar behaviors observed in Figures 3B, 6A imply that the requirement for the maximization of the mutual information entropy affects the distribution of the connectivity strength *w*_*ij*_, which converges to the model, indicating the critical state. These results support the argument that the criticality of the connectivity strength has its origin in that of the information transfer.

Thus, the maximization of the mutual information entropy explains the origin of the phase transition in the topology and the criticality in the coactivation patterns. Although these two important attributes of the brain are established separately, they are directly related by the maximization. These findings provide a unified perspective for self-organized criticality and small-world topology, under the mechanism driven by the maximization of the mutual information entropy.

### Biological constraint

Our findings also reveal the contribution of the biological constraints to the mechanism regulating the information transfer. We had specified the critical point by the sharp peaks in Figures 3B, 4, and 6B. In these figures, the vertical axis, the largest component size ratio in the network, indicates that, at this point, the network structure shows the phase transition from the fully connected state to the fragmented one, which contains isolated components (Takagi, 2017). This state is relevant for maintaining the brain activity, because the fully connected structure might allow the integration of the signals (Tononi et al., 1994; Bassett and Bullmore, 2006; Bassett et al., 2006; Kitzbichler et al., 2009; Sporns, 2013) from functionally specialized regions in the brain (Tononi et al., 1994; Hilgetag and Grant, 2000; Sporns, 2013). Therefore, this state, the fully connected network, might be a minimum requirement for the integrated function of the brain (Tononi et al., 1994; Bassett and Bullmore, 2006; Hagmann et al., 2008; Sporns, 2013), and our result suggests that the brain network satisfies this constraint.

On the other hand, the same set of Figures 3B, 4, and 6B, indicate that this criticality is obtained by reducing the excess connections under the above constraint of integration. At this critical point, the integrated structure with the fully connected topology is preserved with the minimal connections, because the lower threshold allows excess connections. From the point of view of economic expenditure of energy (Achard and Bullmore, 2006; Bassett and Bullmore, 2006; Chen et al., 2006; Bassett et al., 2010; Bullmore and Sporns, 2012), suppressing excess connections reduces the energy cost of the network wiring and the biological energy consumption associated with the activity. Our results imply that the cost-effective state, without losing its function, is realized at this critical point (Takagi, 2017). We suggest that requirements for reducing the energy consumption and preserving the integrated state in the brain network work as the biological constraints to determine the optimal state of the brain network.

### Concluding remarks

The results from this study provide evidence to support the argument that the brain network is optimized with regard to information processing. This study suggests the principle and the constraint required for the mechanism underlying the information transfer in the brain network. Our results specifically suggest that, under the constraint of preserving the fully connected network structure, reducing the energy consumption and maximizing the information transfer are the principles governing the topological and functional aspects of the brain network. Thus, our results provide an insight into the mechanism of information processing in the brain.

Based on the simulation presented here, we describe the dynamics of the brain network in response to activation probability and the connectivity threshold, which are the major factors affecting mutual information entropy. Our conclusion is consistent with empirical data, such as those obtained regarding small-world topology and the criticality of the brain network. These findings are widely supported by various experimental and simulation results. Yet, how the requirement for optimization in information processing affects network developments in real brains remains unknown.

## Author contributions

KT designed the study, conducted the simulations and data analyses, and wrote the manuscript.

### Conflict of interest statement

The author declares that the research was conducted in the absence of any commercial or financial relationships that could be construed as a potential conflict of interest. The reviewer, YL and the handling Editor declared their shared affiliation
